# Intra-Articular Injection of Stromal Cell-Derived Factor 1α Promotes Meniscal Healing via Macrophage and Mesenchymal Stem Cell Accumulation in a Rat Meniscal Defect Model

**DOI:** 10.3390/ijms21155454

**Published:** 2020-07-30

**Authors:** Yohei Nishida, Yusuke Hashimoto, Kumi Orita, Kazuya Nishino, Takuya Kinoshita, Hiroaki Nakamura

**Affiliations:** Department of Orthopaedic Surgery, Osaka City University Graduate School of Medicine, Osaka 545-8585, Japan; special_week_5@hotmail.com (Y.N.); m1294646@msic.med.osaka-cu.ac.jp (K.O.); kazuya_best@hotmail.com (K.N.); zidane595@gmail.com (T.K.); hnakamura@med.osaka-cu.ac.jp (H.N.)

**Keywords:** SDF-1, meniscus, macrophage, mesenchymal stem cell

## Abstract

The stromal-cell-derived factor-1α (SDF-1) is well-known for playing important roles in the regeneration of tissue by enhancing cell migration. However, the effect of SDF-1 in meniscal healing remains unknown. The purpose of this study is to investigate the effects of intra-articular injection of SDF-1 on meniscus healing in a rat meniscal defect model. The intra-articular SDF-1 injection was performed at meniscectomy and one week later. Macroscopic and histological assessments of the reparative meniscus were conducted at one, two and six weeks after meniscectomy in rats. In the macroscopic evaluation, the SDF-1 group showed an increase in the size of the reparative meniscus at six weeks after meniscectomy compared to the phosphate-buffered saline (PBS) injection (no-treatment) group. Histological findings showed that intra-articular injection of SDF-1 enhanced the migration of macrophages to the site of the regenerative meniscus at one and two weeks after meniscectomy. CD68- and CD163-positive cells in the SDF-1 group at one week after meniscectomy were significantly higher than in the no-treatment group. CD163-positive cells in the SDF-1 group at two weeks were significantly higher than in the no-treatment group. At one week after meniscectomy, there were cells expressing mesenchymal-stem-cell-related markers in the SDF-1 group. These results indicate the potential of regenerative healing of the meniscus by SDF-1 injection via macrophage and mesenchymal stem cell accumulation. In the present study, intra-articular administration of SDF-1 contributed to meniscal healing via macrophage, CD90-positive cell and CD105-positive cell accumulation in a rat meniscal defect model. The SDF-1–CXCR4 pathway plays an important role in the meniscal healing process. For potential clinical translation, SDF-1 injection therapy seems to be a promising approach for the biological augmentation in meniscal injury areas to enhance healing capacity.

## 1. Introduction

A meniscus is a crescent-shaped fibrocartilage tissue in the knee joint [1]. It is an important structure that acts as a shock absorber, lubricant and stabilizer of the knee [2]. Injured menisci cause early changes in the boundary-lubricating ability of synovial fluid and in the integrity of articular cartilage [3]. Compared with normal menisci, degenerated menisci contain decreased amounts of extracellular matrix components, such as lubricin, type II collagen and aggrecan [4]. Arthroscopic partial meniscectomy is commonly performed on patients with meniscus injuries and degeneration [5] because of poor healing and limited blood supply. However, subtotal or partial meniscectomy has been reported to be a potent risk factor for osteoarthritis (OA) [6]. Hence, it is important to enhance meniscal healing of injured or degenerated menisci to prevent OA progression.

Stromal-cell-derived factor-1 (SDF-1) is a chemokine also known as C-X-C motif chemokine ligand 12. SDF-1 is a potent chemotactic cytokine with various biological functions, such as inflammatory cell infiltration, angiogenesis and stem cell migration [7,8]. It has been reported to play important roles in the regeneration of tissue, including in the heart [9,10], brain [11], liver [12] and kidney [13]. However, to our knowledge, only a few studies have clarified the effect of SDF-1 in meniscal healing.

Several studies have investigated the potential of different strategies for enhancing meniscal healing, including the use of growth factors [14], stem cells [15] and the application of artificial scaffolds [16] in vivo. The joint environment is more complex because of the presence of many different cells, tissues and inflammatory factors produced in the joint following meniscal injury [14]. Cell-based strategies entail a two-step surgery, as they require cell harvesting and seed cell expansion in vitro prior to transplantation [17]. As SDF-1 directs multiple cells derived from bone marrow to areas of tissue damage and enhances regeneration [18], administration of SDF-1 can be expected to directly promote meniscal healing. Additionally, intra-articular injection of SDF-1 can be performed directly in an operating room. It is a one-step surgery that can be performed without additional testing steps while reducing costs and risks.

We previously reported that meniscus healing involved macrophage (CD68^+^, CD163^+^) accumulation, the same as a wound-healing mechanism [19,20]. Hence, we hypothesized that the intra-articular injection of SDF-1 to the injured site could enhance repair of the meniscus by promoting meniscal differentiation through macrophage and mesenchymal stem cell (MSC) accumulation. The specific objectives of this study were to clarify the effects of SDF-1 by intra-articular injection at the injury site of the meniscus.

## 2. Results

### 2.1. Intra-Articular Injection of SDF-1 Promoted Meniscal Repair

**Figure 1:**
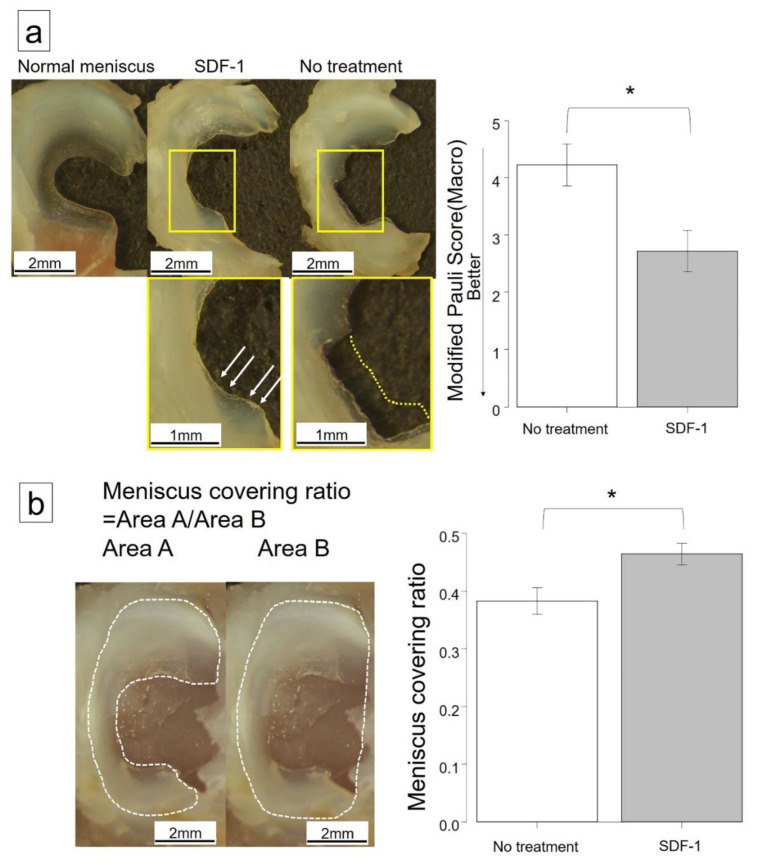
Macroscopic examination of regenerated meniscus at 6 weeks postoperatively. (**a**) Macroscopic observation. The stromal-cell-derived factor-1 (SDF-1) group appeared more regenerated at 6 weeks after meniscectomy (white arrows). The yellow dotted line shows the edge of the original meniscus. Pauli’s macroscopic score was significantly lower in the SDF-1 group than in the no-treatment group (2.71 vs. 4.22, *p* = 0.026). (**b**) Explanation for meniscus covering ratio, defined as the ratio of medial meniscus area (white dots of Area A) to medial plateau area (white dots of Area B). The meniscus covering ratio was larger in the SDF-1 group than in the no-treatment group at 6 weeks after meniscectomy (0.48 vs. 0.37, *p* = 0.018). Bars represent the mean ± SD (*n* = 7). * *p* < 0.05.

Compared with the no-treatment group, the SDF-1 group increased the size of the regenerated meniscus at six weeks after meniscectomy (Figure 1a). In the quantitative evaluation using Pauli’s macroscopic score [21], the SDF-1 group was significantly lower than the no-treatment group (2.71 vs. 4.22, *p* = 0.026). The meniscus covering ratio (Figure 1b) [22] was larger in the SDF-1 group than in the no-treatment group at six weeks after meniscectomy (0.48 vs. 0.37, *p* = 0.018).

**Figure 2:**
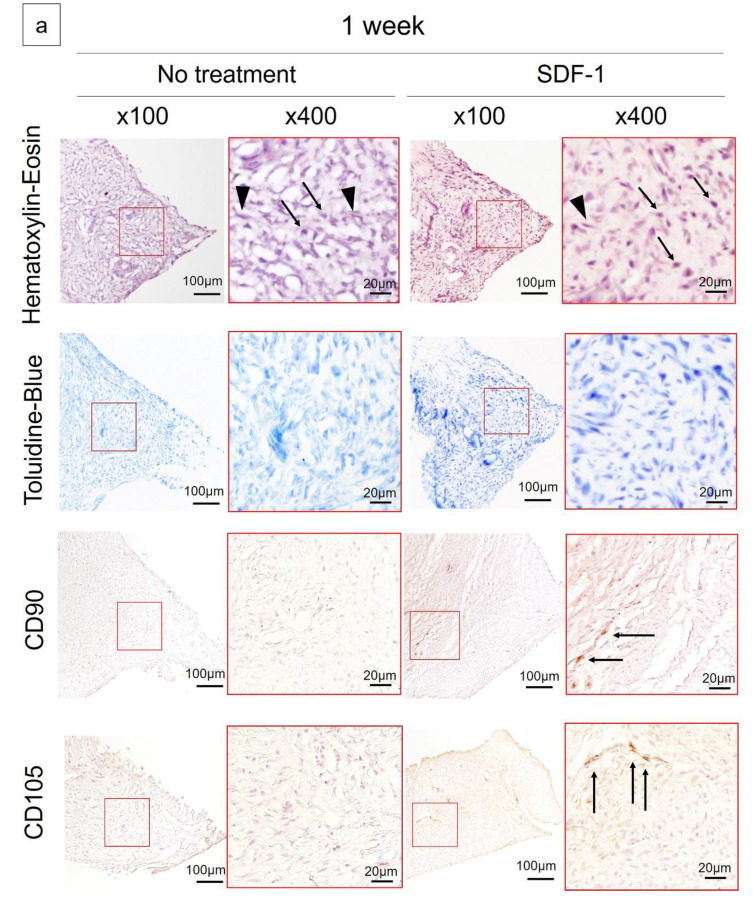

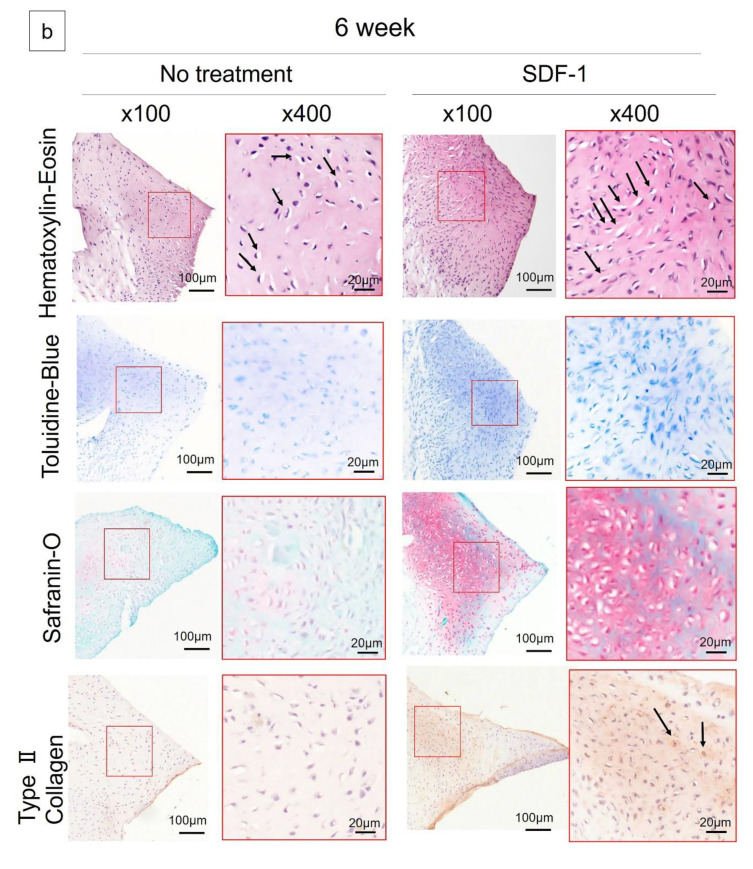

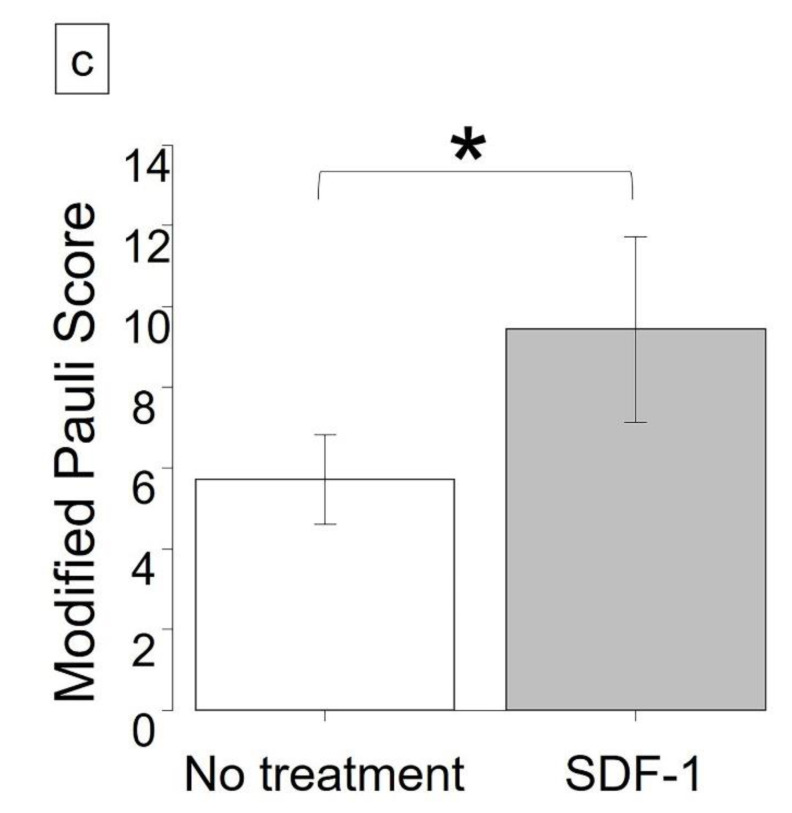
(**a**) Histological findings 1 week after meniscectomy. Hematoxylin and eosin (H–E) staining of the reparative meniscus revealed a mixture of mononuclear cells (arrows) and spindle-type cells (arrowheads). Toluidine blue staining revealed no metachromasia in either group. Immunohistochemistry revealed a few CD90- and CD105-positive cells only in the reparative tissue of the stromal-cell-derived factor-1 (SDF-1) group. (**b**) Histological findings 6 weeks after meniscectomy. H–E staining revealed cells with lacunae (arrows), indicative of fibrochondrocyte-like cells. Toluidine blue staining revealed more metachromasia in the SDF-1 group. Safranin-O-stained reparative tissue in the SDF-1 group. Immunohistochemistry exhibited positive staining of type II collagen in the cells (arrows) and surrounding extracellular matrix in the SDF-1 group but not in the no-treatment group. Scale bars indicate 100 μm in the 100× magnified images and 20 μm in the 400× magnified images. (**c**) A modified Pauli’s scoring system was used to quantify the regeneration of the meniscus; the SDF-1 group was higher than the no-treatment group (9.42 vs. 5.71, *p* = 0.01). * *p* < 0.05.

Histologically, in hematoxylin–eosin (H–E) staining, a mixture of round and spindle-shaped cells was observed in the regenerated meniscus one week after meniscectomy in both groups. Toluidine blue staining revealed no metachromasia in either group. Immunohistochemistry revealed a few CD90- and CD105-positive cells only in the reparative tissue of the SDF-1 group at one week (Figure 2a). At six weeks after meniscectomy, the repaired tissue sections of both groups appeared triangular in shape. H–E staining revealed cells with lacunae, indicative of fibrochondrocyte-like cells, in both groups (Figure 2b). Toluidine blue staining revealed metachromasia in both groups; however, the staining was stronger in the SDF-1 group. A safranin-O-positive area in the regenerated meniscus was observed in the SDF-1 group but not in the no-treatment group. Type II collagen staining in the areas of regeneration was observed in the SDF-1 group but not in the no-treatment group. A modified Pauli’s scoring system [22,23] was used for quantifying the regeneration of the meniscus; the SDF-1 group had greater regeneration than the no-treatment group (9.42 vs. 5.71, *p* = 0.01) (Figure 2c).

### 2.2. SDF-1 Enhances Migration of Macrophages to Injury Site

**Figure 3:**
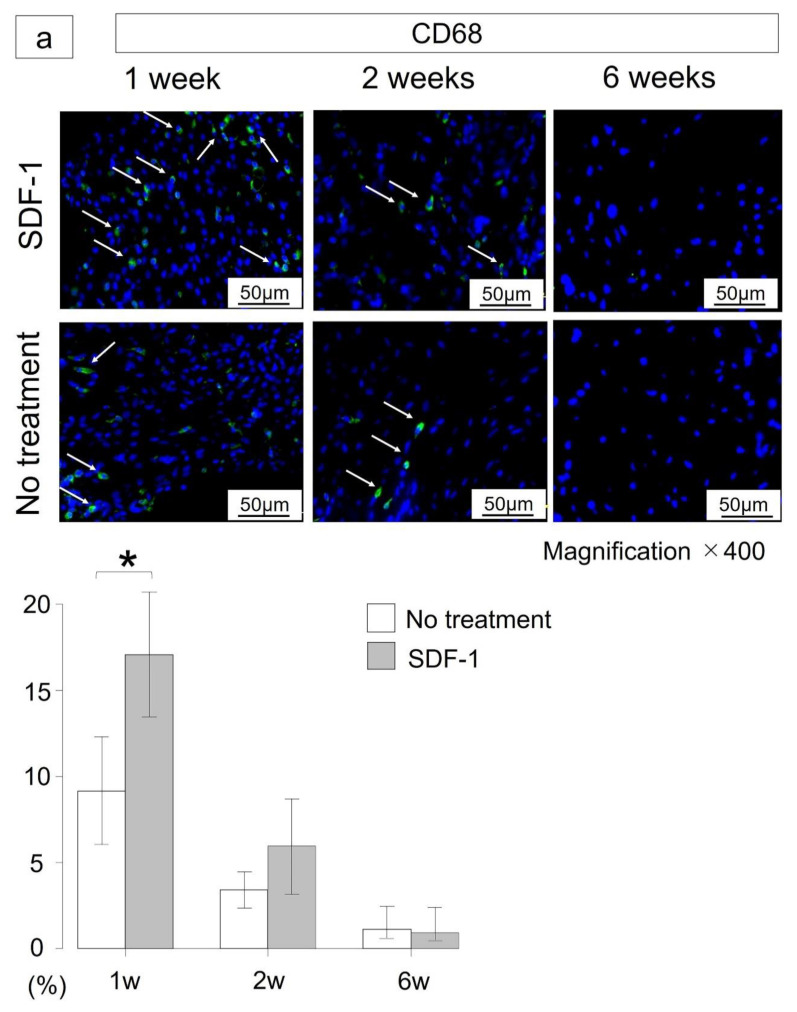

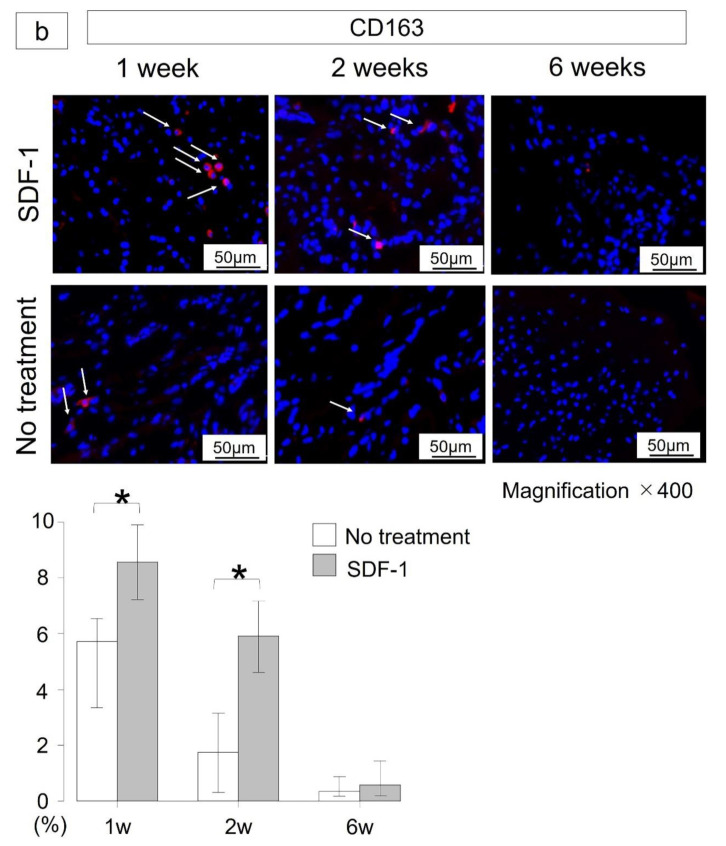

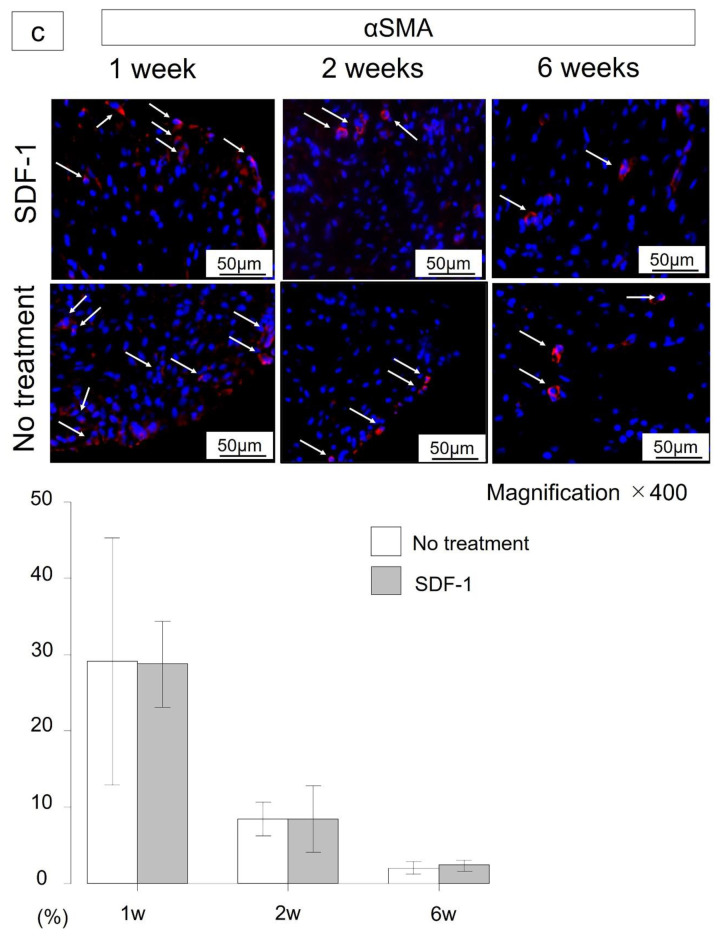
Immunohistochemical examination 1, 2 and 6 weeks after meniscectomy. Blue fluorescence is indicative of 4′,6-diamidino-2-phenylindole (DAPI). Scale bar indicates 50 μm. (**a**) Immunofluorescent staining of CD68 for regenerated meniscus. Green fluorescence indicates CD68 (arrows). At 1 week after meniscectomy, CD68-positive cells in the stromal-cell-derived factor-1 (SDF-1) group were significantly higher than in the no-treatment group (CD68: 9.1% vs. 17.7%, *p* = 0.03). There was no significant difference between both groups at 2 and 6 weeks (*p* = 0.421, 0.737). (**b**) Immunofluorescent staining of CD163 for regenerated meniscus. Red fluorescence indicates CD163 (arrows). CD163-positive cells in the SDF-1 group were significantly higher than in the no-treatment group at 1 week after meniscectomy (8.6% vs. 5.7%, *p* = 0.04). CD163-positive cells in the SDF-1 group were still significantly higher than in the no-treatment group (5.9% vs. 1.7%, *p* = 0.015) at 2 weeks after meniscectomy. At 6 weeks after meniscectomy, the percentage of positive cells decreased to approximately 1%. (**c**) Immunofluorescent staining of α-smooth muscle actin (αSMA) for regenerated meniscus. Red fluorescence indicates αSMA (arrows). There was no significant difference between both groups throughout the study. White bar: no-treatment group. Gray bar: SDF-1 group. Bars show the mean ± SD (n = 5 at 1 and 2 weeks, n = 7 at 6 weeks). * *p* < 0.05.

To assess the effect of the SDF-1 injection on CD68^+^, CD163^+^ and α-smooth muscle actin (αSMA)^+^ cells, each cell’s number was estimated. One week after meniscectomy, CD68-positive and CD163-positive cells in the SDF-1 group were significantly higher than in the no-treatment group (CD68: 9.1% vs. 17.7%, *p* = 0.03; CD163: 8.6% vs. 5.7%, *p* = 0.04) (Figure 3a,b). For αSMA-positive cells, there were no significant differences between both groups (29.1% vs. 28.7%, *p* = 0.84) (Figure 3c). Two weeks after meniscectomy, CD163-positive cells in the SDF-1 group were significantly higher than in the no-treatment group (5.9% vs. 1.7%, *p* = 0.015). For CD68-positive cells and αSMA-positive cells, no significant difference was noted between the two groups (3.4% vs. 5.2% and 8.4% vs. 8.4%, respectively). At six weeks after meniscectomy, the percentage of CD68-positive cells and CD163-positive cells decreased to approximately 1%.

## 3. Discussion

This study revealed that intra-articular injection of SDF-1 promoted the migration of MSC-related markers and CD68- and CD163-positive macrophages at one week and a CD163-positive macrophage at two weeks in a rat meniscal defect model. It resulted in an improvement of the macroscopic and histological findings of reparative meniscal tissues at six weeks by SDF-1 intra-articular injection.

It was reported that vascularity at the injured site strongly influences the outcome of meniscus healing [24]. Additionally, an increased healing rate has been reported when concomitant meniscal repair and anterior cruciate ligament (ACL) reconstruction are performed [25,26]. It is thought that the release of growth factors and various cells for healing after bone tunnel drilling enhances the biological environment at the repair site [27]. We have previously reported that macrophages recruited from bone marrow are also involved at an early stage of meniscal healing, and αSMA-positive cells were migrated into the injured meniscal surface and are essential for integration into adjacent meniscal tissue [20]. Therefore, the mechanism of meniscal healing may be similar to wound healing, which has three stages: inflammation, proliferation and remodeling [28,29]. In the inflammatory phase, neutrophil infiltration is followed by circulating peripheral blood cells and macrophages. Proinflammatory M1 macrophages express CD68, and anti-inflammatory/proregenerative M2 macrophages express antigens such as CD163 [30]. M1 macrophages produce proinflammatory cytokines such as TNFα, IL-6 and IL-12 [31,32,33]. In the proliferative phase, M2 macrophages contribute to tissue healing, angiogenesis, promotion of tumor growth and immunosuppression [31]. M2 macrophages produce high amounts of IL-10 and TGF-β [34,35], promote tissue repair and wound healing and possess proangiogenic properties. Hence, meniscal differentiation may come from various proliferative cytokines derived from M2 macrophages.

Previously, SDF-1 was shown to attract a myriad of cell populations, such as monocytes, lymphocytes and hematopoietic and bone marrow mesenchymal stem cells (BMSCs), to the injury site [7,8]. In this study, we found that the MSC-related marker positive cells were located in the reparative tissue upon SDF-1 treatment. We previously reported the origin and distribution of macrophages during meniscal reparative change [20]. The majority of peripheral blood cells at the early phase of meniscal reparative change were CD68- or CD163-positive macrophages. Here, the proportion of CD68- and CD163-positive macrophages increased in the SDF-1 group compared to the no-treatment group at one week after meniscectomy. The percentage of CD163-positive macrophages remained higher than in the no-treatment group at two weeks after meniscectomy. This indicates that SDF-1 promoted macrophage polarization toward the M2 phenotype by increasing the number of CD163-positive cells. Krieger et al. have demonstrated that SDF-1 can not only induce stem cells but also anti-inflammatory monocytes related to cell homing [18]. Our study is consistent with this result. Therefore, it may indicate that SDF-1 administration increased cell mobilization from the bone marrow in the healing process of meniscal regeneration.

Previously, many studies have reported that intra-articular injection of cells is an effective treatment strategy for regenerating an injured meniscus. Murphy et al. found that local delivery of adult MSCs to injured knee joints could stimulate the regeneration of meniscal tissue [36]. Horie et al. reported that the injected stem cells differentiated directly into meniscal cells within the meniscal defect [15]. However, using the cell approach requires at least two surgeries and can cause complications such as cell contamination, cell differentiation and disease transmission [17,37]. Cell-free strategies do not use cell cultures, and the treatment can be performed as a one-step surgery [37].

MSCs have been identified as both CD90- and CD105-positive cells [38]. Wang et al. reported that SDF-1 promotes periodontal tissue regeneration via CD90-positive stromal cell recruitment [39]. Pernas et al. reported that SDF-1 could recruit CD105-positive MSCs [40]. In this study, we observed CD90- and CD105-positive cells at one week after meniscectomy. This may be due to SDF-1 promoting tissue repair cell migration through the accumulation of MSCs, which resulted in enhanced meniscal repair. The results obtained from this study will be helpful in developing a technique for new strategies for using SDF-1 to promote meniscal healing, which is a safe and minimally invasive approach.

The study has several limitations. First, the concentration of SDF-1 at the injured site could not be measured, and we did not evaluate the variation of the effect due to the concentration. Second, we proved that accumulation of M2 macrophages were increased by intra-articular injection of SDF-1. However, the amount of cytokines produced by M2 macrophages, such as IL-10 or TGF-β, was not measured. Third, we did not prove where these effective cells come from because a parabiosis model was not used in this study. As a previous study showed that macrophages and MSCs were migrated into the injured site from bone marrow, these cells would be suspected to come from bone marrow. Fourth, double staining of CD90- and CD105-positive cells was not examined in this study. A previous study demonstrated that CD90-positive cells play a crucial role in allowing cells to differentiate into multiple cell phenotypes [41]. Additionally, the CD90^+^ subpopulation of synovial fluid mesenchymal progenitor cells has a higher chondrogenic potential compared to that of the CD90- population [42]. Fan et al. reported that CD105 enhances chondrogenesis through Smad2 activation [43]. CD90- and CD105-positive cells may be MSCs and may have contributed to meniscal regeneration in our study. Fifth, we did not perform a dose-dependent study. Chen et al. reported that MSC migration is mediated by SDF-1 in a dose-dependent manner [44]; the dose of SDF-1 was 200 ng/mL at baseline and one week later for bone fractures. Therefore, in this study, the SDF-1 dose was administered at 200 ng to the knee joint at meniscectomy and one week later. Further research is needed to determine the optimal dose for meniscal healing.

## 4. Materials and Methods

### 4.1. Animals

Thirty-four female Lewis rats (eight weeks old) were obtained from Nihon SLC (Shizuoka, Japan). The animals were fed in cages with free access to food and water in an air-conditioned environment. All animal procedures were approved and conducted in accordance with the regulations of the Osaka City University Graduate School of Medicine Committee on Animal Research (approval no. 11002, date of approval: 10 August 2011).

### 4.2. Meniscectomy Model

**Figure 4:**
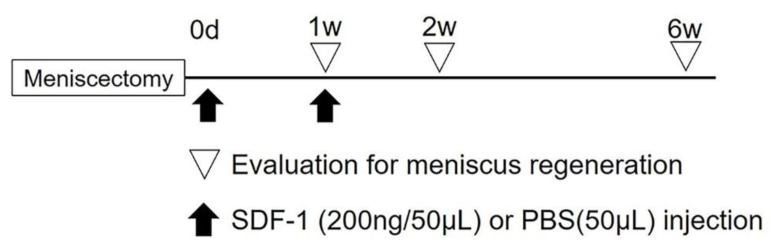
Study protocol. At the time of meniscectomy, 200 ng of SDF-1 with 50 μL of PBS or 50 μL of PBS was administrated, and 5 rats from each group were sacrificed via CO_2_ inhalation 1 week later. The remaining rats in each group were reinjected with 200 ng/50 μL of SDF-1α (or PBS) into the left knee joint at 1 week after meniscectomy. They were then sacrificed via CO_2_ inhalation at 2 and 6 weeks after meniscectomy.

The animals were anesthetized via subcutaneous injection of ketamine (50 mg/mL; Sankyo, Tokyo, Japan) and xylazine (0.2 mg/mL; Bayer HealthCare, Tokyo, Japan) at a ratio of 10:3 and a dose of 1 mL/kg body weight, as previously described [17,18]. A straight incision was made on the anterior side of the left knee. Using the medial parapatellar surgical approach, the knee joint was exposed, and the patella was dislocated laterally. The anterior part of the medial meniscus was cut vertically at the level of the medial collateral ligament, and the anterior half of the medial meniscus was resected. Thereafter, the capsule was immediately closed using 6–0 nylon sutures. Next, 200 ng/50 μL of recombinant SDF-1α (PeproTech Inc., Rocky Hill, NJ, USA) was injected into the left knee joint in the SDF-1 groups (*N* = 17). In the no-treatment groups (*N* = 17), 200 ng/50 μL of PBS (phosphate-buffered saline) instead of SDF-1α was injected into the left knee. Thereafter, skin incisions were closed using 5–0 nylon sutures. Rats were allowed to walk freely in their cages after surgery. One week postsurgery, five rats from each group were sacrificed via CO_2_ inhalation. The remaining rats in each group (*N* = 12, respectively) were reinjected with 200 ng/50 μL of SDF-1α (or PBS) into the left knee joint. They were then sacrificed via CO_2_ inhalation at two (*N* = 5) and six (*N* = 7) weeks after partial meniscectomy. The study protocol is shown in Figure 4.

### 4.3. Macroscopic Observation

The tibial plateau with medial reparative menisci was carefully separated from the femoral condyle at six weeks after meniscectomy. Macroscopic pictures of the meniscus with the tibia were taken using a stereomicroscope (SMZ1500, Nikon, Tokyo, Japan). Quantification of the size of the regenerated meniscus was performed using ImageJ software (version 1.46, National Institutes of Health, Bethesda, MD, USA) to measure the ratio of the whole area of the medial meniscus, including both the regenerated region and normal region, to the whole area of the medial tibial plateau [21]. Thereafter, the meniscus was separated from the tibia, and pictures were also taken. For quantification of macroscopic characteristics of the regenerated meniscus, we used Pauli’s macroscopic scoring system [22].

### 4.4. Histological Examination

The reparative meniscus at one and two weeks after meniscectomy was carefully removed from the femur and tibia. The meniscus at six weeks after meniscectomy was also used for histological examination after photography for macroscopic evaluation. Regenerated meniscal tissues were fixed in 4% paraformaldehyde for 24 h and dehydrated in 10% sucrose for 4 h, 20% sucrose for 4 h and 30% sucrose overnight at 4 °C. Thereafter, tissues were frozen and stored in the dark at −80 °C after being embedded in optimal cutting temperature (OCT) compound (Sakura Finetek, Torrance, CA, USA). Specimens were cut radially at 5 µm thickness using a cryostat. Specimens from one week after meniscectomy underwent immunofluorescence staining, and those from six weeks were stained with hematoxylin and eosin, toluidine blue or safranin O and Fast Green for histological evaluation of cell morphology and meniscus regeneration. For hematoxylin and eosin staining, sections of the harvested tissues were rinsed in PBS for 5 min at room temperature (RT, 20 °C), and then the slides were stained with Mayer’s hematoxylin (Muto Pure Chemicals Co. Ltd., Tokyo, Japan) for 10 min, and after being rinsed in PBS, the slides were stained with eosin (1% Eosin Y, Muto Pure Chemicals Co. Ltd.) for 10 min. For toluidine blue staining, sections of the harvested tissues were rinsed in PBS for 5 min at RT, and then the slides were stained with toluidine blue solution (0.05% toluidine blue pH 4.1, Muto Pure Chemicals Co. Ltd.) for 15 min. For safranin O and Fast Green staining, sections of the harvested tissues were rinsed in PBS for 5 min at RT, and then the slides were stained with 0.1% Fast Green (ACROS Organics 2353-45-9, Fast Green FCF, Thermo Fisher Scientific K.K., Tokyo, Japan) for 5 min and stained with 0.1% safranin O (ACROS Organics 477-73-6, Safranin O, Thermo Fisher Scientific K.K.). In all stains, the material was dehydrated with ethanol and sealed after permeation with xylene. Microscopic evaluation was performed using the Olympus BX53 (Olympus, Tokyo, Japan). The regenerated meniscus at six weeks after meniscectomy was evaluated using the quantitative score based on the modified Pauli’s score [23], on a scale of 0–15. The Pauli score included tissue surface characteristics, cellularity, matrix and collagen fiber organization and safranin-O–Fast Green matrix staining intensity [22]. The modified Pauli score excluded the surface of the inner border from the original method [23].

### 4.5. Immunohistochemistry

CD90 and CD105 staining was performed to confirm the presence of MSC for the meniscus at one week after meniscectomy, and type II collagen staining was performed to confirm the presence of type II collagen in the reparative meniscus six weeks after meniscectomy in the SDF-1 and no-treatment groups, respectively. Immunohistochemistry staining was performed with reference to the previously reported method [19,45]. To prepare for immunohistochemistry staining, sections of harvested tissue embedded in OCT were rinsed three times (5 min each time) in PBS to remove the OCT compound. Slides were incubated with citrate buffer (S1699, Target Retrieval Solution, 10×, DAKO Japan, Tokyo, Japan) in PBS for 20 min at RT for optimal antigen retrieval. Endogenous peroxidases were quenched using 1.0% hydrogen peroxidase in methanol for 30 min at RT. Slides were then rinsed with PBS and incubated with 10% goat serum for 1 h at RT. Thereafter, specimens were incubated with anti-CD90 primary antibody (dilution 1:200; ab133350, Abcam, Cambridge, MA, USA), anti-endoglin (CD105) antibody (dilution 1:100; 05–1424, Merck Millipore, Burlington, MA, USA) or anti-type II collagen primary antibody (dilution 1:200; mouse IgG fraction, Kyowa Pharma Chemical Co. Ltd., Takaoka, Japan) for 1 h at room temperature (RT, 20 °C). Specimens were washed three times with PBS for 10 min, and slides were incubated with a peroxidase-labeled antibody (Histofine Simple Stain, Nichirei Biosciences Inc., Tokyo, Japan) for 1 h at RT. After specimens were washed three times with PBS for 10 min, the immunoreaction was visualized by incubating the sections for 3 min in 3,3′-diaminobenzidine (Histofine Simple DAB solution, Nichirei Biosciences Inc.). The sections were lightly counterstained with Mayer’s hematoxylin (Muto Pure Chemicals Co. Ltd.) and mounted (Permount, Falma, Tokyo, Japan). Microscopic evaluation was performed using the Olympus BX53 (Olympus).

### 4.6. Immunofluorescence Staining of CD163, CD68 and αSMA

The regenerated meniscus at one, two and six weeks after meniscectomy was used for evaluation. Immunofluorescence staining was performed as previously reported [20]. In preparation for immunofluorescence staining, sections of harvested tissue embedded in OCT were rinsed three times (5 min each time) in PBS to remove the OCT compound. Slides were incubated with citrate buffer (S1699, Target Retrieval Solution, 10×, DAKO Japan, Tokyo, Japan) in PBS for 20 min at RT for optimal antigen retrieval. Endogenous peroxidases were quenched using 1.0% hydrogen peroxidase in methanol for 30 min at RT. Slides were rinsed once more with PBS and incubated with 10% goat serum for 1 h at RT. The primary antibody was added and incubated overnight at 4 °C. Rabbit polyclonal anti-CD68 antibodies were utilized to identify proinflammatory macrophages (dilution 1:400; ab125212, Abcam, Cambridge, MA, USA). Mouse monoclonal anti-CD163 and anti-αSMA antibodies were utilized to identify tissue-repairing macrophages (dilution 1:500; MA342R, AbD SeroTec, Oxford, UK) and myofibroblasts (dilution 1:400; A2547, Sigma-Aldrich, St. Louis, MO, USA). Following washing three times with PBS/0.1% Tween-20 for 10 min, sections stained with CD68 were incubated with goat anti-rabbit Alexa Fluor 647 secondary antibody (dilution 1:500; ab150079, Abcam) and then stained with CD163 and αSMA and incubated with goat anti-mouse Alexa Fluor 594 secondary antibody (dilution 1:500; A-11032, Invitrogen, Eugene, OR, USA) for 1 h at RT. After washing three times with PBS for 10 min, stained sections were mounted with VECTASHIELD Mounting Medium (Vector Laboratories, Burlingame, CA, USA) containing 4′,6-diamidino-2-phenylindole (DAPI; Sigma-Aldrich, St. Louis, MO, USA) to stain nuclei. During this process, slides, including the apparatus, were covered with foil sheets to protect samples from photodamage. Images were acquired using an all-in-one fluorescence microscope (BZ-X800, Keyence, Osaka, Japan).

### 4.7. Fluorescent Microscopic Examination

For immunofluorescent quantification, DAPI and specific markers of interest were counted from each meniscal section in three randomly selected areas under high power (400× magnification). To determine the proportion of cells stained positive by primary antigens, the following formula for the positive cell rate was used: (X, DAPI-double-positive cell population/DAPI-positive cell population) × 100 (%), where X = CD68, CD163 or αSMA.

### 4.8. Statistical Analysis

The Mann–Whitney U test was used for all comparisons between the SDF-1 and no-treatment groups. This included the Pauli score, meniscus covering ratio and modified Pauli score at six weeks after meniscectomy as well as CD68-, CD163- or αSMA-positive cell rates at one, two and six weeks after meniscectomy. Analysis was performed with the R (version 3.5.1, patched, http://www.r-proje ct.org, The R Foundation, Vienna, Austria) software package, and significance was assumed at *p* < 0.05.

## 5. Conclusions

In the present study, intra-articular administration of SDF-1 contributed to meniscal healing via macrophage, CD90-positive cell and CD105-positive cell accumulation in a rat meniscal defect model. The SDF-1–CXCR4 pathway plays an important role in the meniscal healing process. For potential clinical translation, SDF-1 injection therapy seems to be a promising approach for biological augmentation in meniscal injury areas to enhance healing capacity.
